# Glucose and unstructured physical activity coupling during sleep and wake in young adults with type 1 diabetes

**DOI:** 10.1038/s41598-022-09728-2

**Published:** 2022-04-06

**Authors:** Stephanie Griggs, Eric Barbato, Estefania Hernandez, Devansh Gupta, Seunghee Margevicius, Margaret Grey, Ronald L. Hickman

**Affiliations:** 1 Ruth M. Anderson Endowed Professor of Nursing and Associate Dean for Research, Frances Payne Bolton School of Nursing, Case Western Reserve University, Cleveland, OH 44106 USA; 2 Department of Genetics and Genome Sciences, School of Medicine, Case Western Reserve University, Cleveland, OH 44106 USA; 3 Department of Anthropology, Frances Payne Bolton School of Nursing, Case Western Reserve University, Cleveland, OH 44106 USA; 4 Department of Population and Quantitative Health Sciences, Case Comprehensive Cancer Center, School of Medicine, Case Western Reserve University, Cleveland, OH 44106 USA; 5 Annie Goodrich Professor of Nursing and Professor of Pediatrics, School of Nursing and School of Medicine, Yale University, West Haven, CT 06477 USA

**Keywords:** Type 1 diabetes, Diabetes complications

## Abstract

Glucose variations have a bidirectional relationship with the sleep/wake and circadian systems in type 1 diabetes (T1D); however, the mechanisms remain unclear. The aim of this study was to describe the coupling between glucose and unstructured physical activity over 168 h in young adults with T1D. We hypothesized that there would be differences in sleep and wake characteristics and circadian variations. Glucose was measured with a continuous glucose monitoring device every 5 min and activity with a non-dominant wrist-worn actigraph in 30-s epochs over 6–14 days. There was substantial glucose and unstructured physical activity coupling during sleep and wake, along with circadian variation based on the wavelet coherence analysis. The extent to which glucose fluctuations result in disrupted sleep over longer than one week should be examined considering the harmful effects on achieving glycemic targets. Further studies are needed to delineate the respective roles of glucose production and utilization and the potential for improved meal and insulin timing to optimize glucose and sleep in this population reliant on exogenous insulin.

Type 1 Diabetes (T1D) is a T cell-mediated autoimmune disease with beta-cell destruction and insulin deficiency^1^. Glucose can fluctuate widely throughout the day and night due to the absent autoregulation in T1D and the shortcomings of insulin therapy^2^. Glucose homeostasis differs between sleep and wake states in individuals with T1D^3–5^. Two processes that regulate sleep (intrinsic circadian timing system and the homeostatic sleep–wake system or state) and state- and circadian-related glucose patterns have been observed over 24 h. Glucose increases in the late afternoon to early evening and rises until the middle of the night^6–9^.

Structured physical activity, a common physiological stressor that perturbs glucose homeostasis and energy needs, leads to hyperglycemia in individuals with T1D and without a chronic condition^10–12^. Two categories of structured physical activity, aerobic (requiring oxygen to generate energy) and anaerobic (activity that breaks down glucose for energy without using oxygen), have divergent effects on blood glucose levels^12^. Structured physical activity over a long duration (e.g., aerobic exercise, 30–90 min with a consistent heart rate of 120–150 beats per minute) leads to a rapid drop in glucose due to the higher glucose disposal rates. In contrast, structured physical activity of short duration and high intensity (e.g., anaerobic exercise, seconds to minutes) leads to a rise in glucose in individuals with T1D^12^.

Brief movements during sleep are strongly coupled to rapid glucose fluctuations that lead to sleep disruption in young adults with T1D^4^. This sleep disruption can negatively impact the ability to achieve glucose targets^13^. Specifically, adults with T1D require more insulin due to consistent shifts between disrupted sleep and sleep compensation^14^. Structured physical activity can change glucose levels several hours later in adults with T1D^15^. Unstructured physical activity (routine continuous activity) exhibits a sleep/wake pattern and may contribute significantly to state- or circadian-related glucose changes^4^.

Elucidating the dynamic bidirectional relationship between glucose and unstructured physical activity changes may help explain their physiological relationship. Although coupling between glucose and routine activity varies over time^4^, there has been limited investigation into the state (sleep–wake) and circadian influences on glucose homeostasis in individuals with T1D, particularly in young adults with T1D who have the lowest rate of achieving glycemic targets compared to adolescents, middle-aged, and older adults with T1D^16^.

Time-varying and frequency-specific coupling between two signals are identified through wavelet coherence analysis (WCA)^17–19^, such as actigraphy power and glucose variations. WCA is an innovative approach that allows one-dimensional time data to be decomposed into the two-dimensional time–frequency domain, rather than the blunt categorizations of short (1 day) and long term (over one week) trends of traditional methods (e.g., general linear model)^18,19^. The specific aims of this investigation were to: (1) quantify the coupling between glucose variations and unstructured physical activity over 168-h (7-days); (2) determine sleep and wake differences in this coupling; and (3) identify circadian variations in glucose/activity coupling in individuals with T1D during young adulthood (18–30 years). We hypothesized that there would be state and circadian differences in the coupling. Therefore, we designed our study to replicate Farabi et al.'s findings over 60 h expanding to 168 h in our study to capture both weekdays and weekend days^4^.

## Results

### Participants

Of the 46 participants who consented and provided questionnaires, 41 wore the continuous glucose monitor (CGM) and Spectrum Plus concurrently for 7 continuous days/nights and were included in this analysis. We excluded 5 individuals who had > 20% missing data (e.g., glucose sampling was every 15 min instead of every 5). We included 7 days of data to be consistent across participants.

We present the clinical and demographic characteristics of the 41 included in the study in Table 1. As highlighted in Table 1, participants' mean HbA1C was slightly higher (7.2 ± 1.1 mg/dL % or 55 mmol/mol), than the target recommended by the American Diabetes Association and comparable to the T1D Exchange cohort currently using CGM technology (HbA1c 7.4 ± 1.0–8.3 ± 1.5% or 57–67 mmol/mol)^16^. Approximately half of the participants were full-time college students (48.8%), 43.9% were working, and 7.3% were not working (4.9% were on leave, and 2.4% were unemployed).

**Table 1:** Demographic and clinical characteristics (N = 41).

	Mean or N	SD or (%)
Age (years)	22.2	3.0
Sex (% female)	27	(65.9)
BMI (kg/m^2^)	26.6	4.4
Epworth sleepiness scale	7.6	3.2
Meal timing breakfast	9:16	1:23
Meal timing lunch	13:16	1:04
Meal timing dinner	19:07	0:58
**Actigraphy**		
Total sleep time (min)	421.5	61.7
Sleep efficiency (%)	85.3	4.9
Sleep onset latency (min)	20.0	13.7
Sleep fragmentation	18.0	5.9
Wake after sleep onset (min)	35.4	17.7
**T1D characteristics**		
T1D duration (years)	10.3	6.1
HbA1C (%)	7.2	1.1
Insulin pump (% yes)	32	(70)
**7-day CGM measures**		
Average	161.6	29.4
SD	59.7	15.9
CV	36.7	5.9
J index	50.7	18.5
MAD (%)	7.9	4.9
Time in range (%)	62.0	15.6
Time above range (%)	33.5	17.2
Time below range (%)	4.4	7.2

*BMI* body mass index, *CGM* continuous glucose monitoring, *SD* standard deviation, *CV* coefficient of variation, *J index* overall quality of glucose variability, *MAD* mean amplitude; time in range (70–180 mg/dL); time above range (> 180 mg/dL); and time below range (< 70 mg/dL).

### Coupling between glucose and activity over the 168-h period

**Figure 1:**
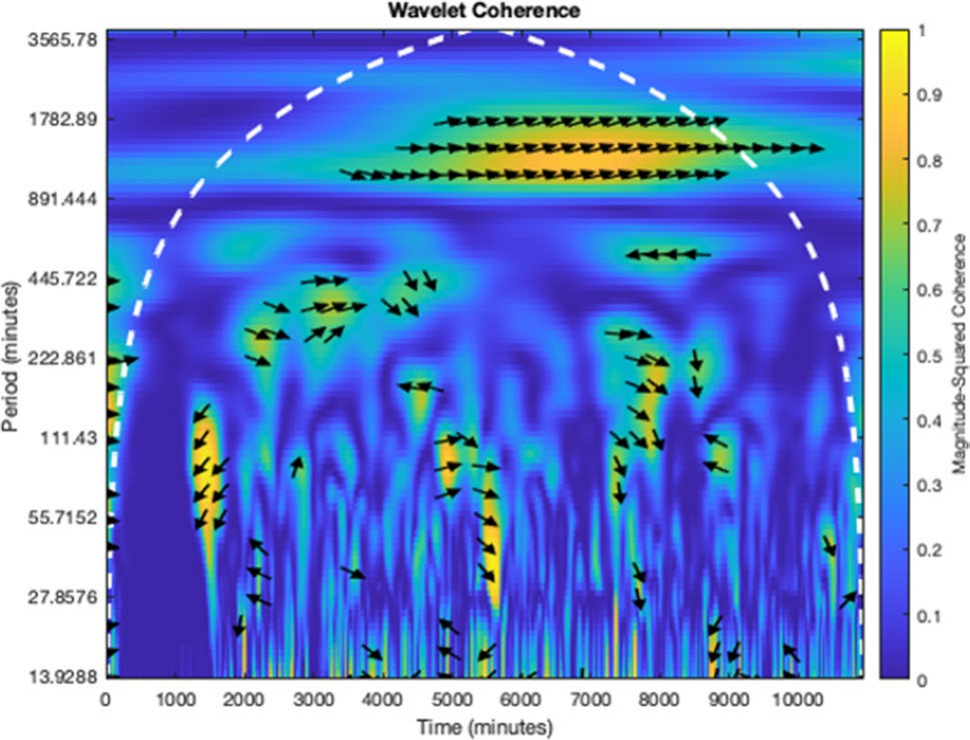
Cross-wavelet transform. Results of cross wavelet transform for the pair of signals *x*(*t*) and *y*(*t*) were obtained using Morlet wavelet with ω_0_ = 2π. Patterns of sleep and glucose were associated with time- and frequency-dependent coherence between these two processes and are represented as a heat map below.

**Figure 2:**
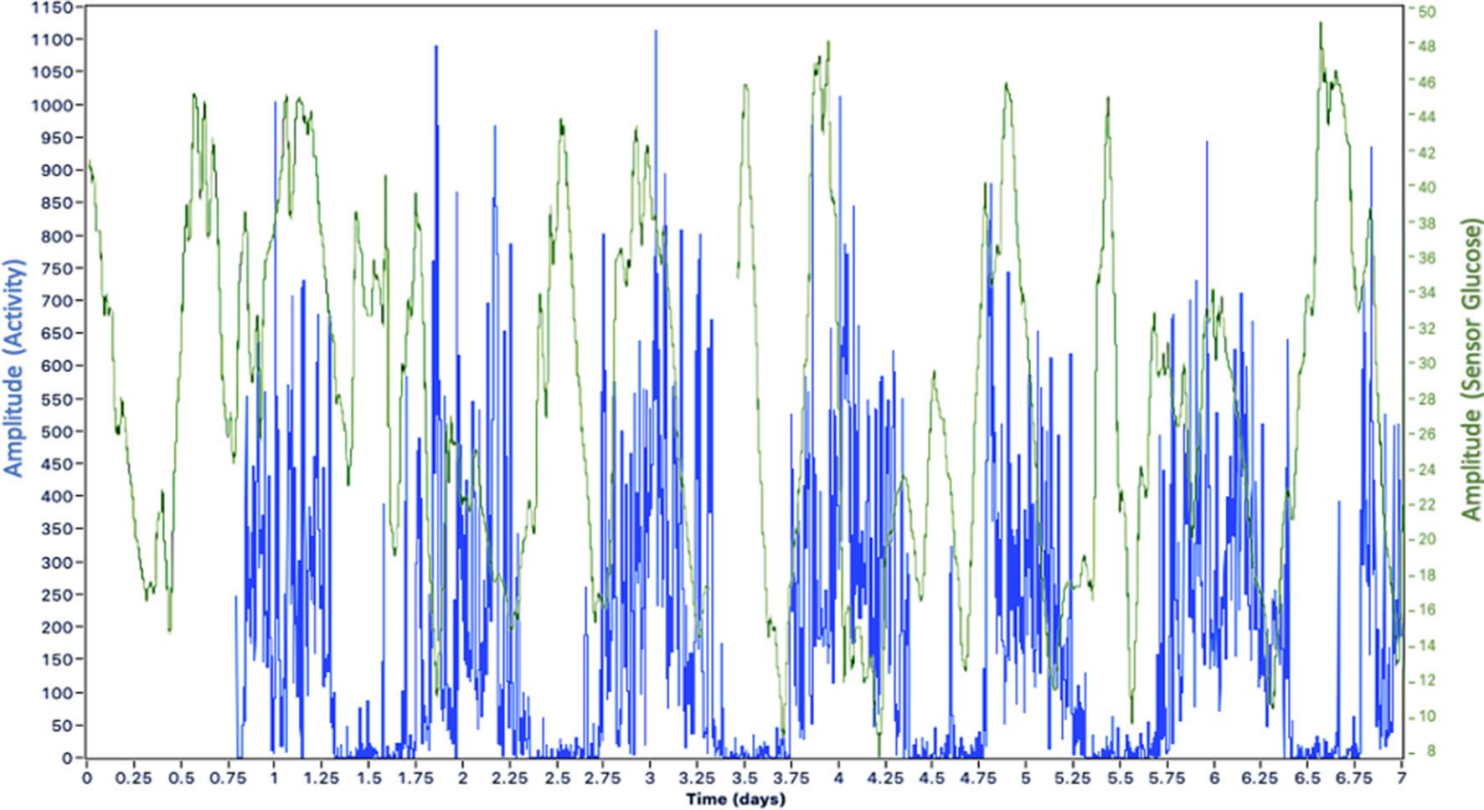
Characteristic patterns of unstructured physical activity and glucose with days and sleep/wake intervals for a single participant. The panel illustrates raw activity (total counts per 5 min [blue]) and glucose values (mean [green]) over a 7-day recording period, with clear daily circadian variations in both activity and glucose.

Figure 1 is a sample from one participant depicting patterns of sleep and glucose and the association with time and frequency-dependent coherence. Arrows pointing east (i.e., right-hand side) reflect an in-phase positive correlation (e.g., both sleep and glucose move in the same direction). Arrows pointing west (left-hand side) reflect an anti-phase–negative correlation. If an arrow is pointed east and downwards, then sleep (A) leads glucose (B), but sleep is lagging glucose if the arrow is pointed upwards. The opposite is true if the arrow is pointing west and upwards, sleep (A) is leading glucose (B) in a negatively correlated situation, but sleep is lagging glucose if an arrow is pointing downwards. Warmer colors (yellow) represent regions with significant interrelation, while colder colors (blue) signify lower dependence between the series or no dependence at all. The white curved dashes indicate the cone of influence (COI), which signifies no statistical confidence and thus should be neglected. We also present characteristic patterns of unstructured physical activity and glucose with days and sleep/wake intervals for a single participant in Fig. 2. The panel illustrates raw activity over a 7-day recording period, with clear daily circadian variations in both activity and glucose.

The group mean coherence over the 168-h interval and overall fluctuation period (10–2880 min) was 0.29 ± 0.09. Mean coherence was lowest for the faster oscillation bands (Bands 1–3) and highest in the slower oscillation bands (Bands 6 and 7). The mean coherence by band was Band 1, 0.25 ± 0.05; Band 2, 0.26 ± 0.06; Band 3, 0.26 ± 0.05; Band 4, 0.28 ± 0.05; Band 5, 0.28 ± 0.07; Band 6, 0.32 ± 0.12, and Band 7, 0.34 ± 0.15.

In the first model (adjusted for sex), mean coherence in bands 1 and 3 were significantly lower than that for Band 7 (*p* < 0.0001*).* Similarly, when adjusting for sex and meal timing (model 2), mean coherence in bands 1 and 3 was significantly lower than that for Band 7 (*p* < 0.0001, *p* = 0.0001, and *p* < 0.0001 for each)*.*

### Differences in coherence between sleep and wake states

In the first model (sex-adjusted), the significant coherence value was higher for slower oscillations (Band 7) than for faster oscillations (Bands 1 to 5) based on the post hoc comparisons with Bonferroni correction (Band 7 vs. Bands 1 to 5 (*p* < 0.0001, *p* < 0.0001, *p* < 0.0001, *p* = 0.0004, and *p* = 0.0008 for each). The mean coherence during the sleep period was greater than the wake period (*p* = 0.0481). The band and sleep/wake state treated as within-person repeated factors explained a significant band effect (*F* = 10.81, *p* < 0.0001) and marginally significant effect of the sleep/wake state (*F* = 4.34, *p* = 0.048) on mean coherence. However, the sleep/wake band interaction effect was not significant.

In the second model (sex and meal timing adjusted), the significant coherence value was higher for slower oscillations (Band 7) than for faster oscillations (Bands 1 to 4) based on the post hoc comparisons with Bonferroni correction (Band 7 vs. Bands 1 to 4 (*p* < 0.0001, *p* − 0.0001, *p* < 0.0001, and *p* = 0.0253, for each). The band and sleep/wake state treated as within-person repeated factors explained a significant band effect (*F* = 9.30, *p* < 0.0001). However, the sleep/wake state and the sleep/wake band interaction effect were not significant. Thus, after adjusting for meal timing the sleep/wake state was no longer significant, but the sleep/wake band interaction effect was not significant in either adjusted model (sex nor meal timing).

### Circadian variations

Coupling between glucose and activity exhibited a circadian pattern. In the first model (sex-adjusted), the mesor for coherence increased from Band 1 through Band 7 (Table 2). There was a significant band effect on the mesor (*F* = 9.99, *p* < 0.0001), and the post hoc comparisons with Bonferroni correction revealed that the Band 7 was significantly greater than Bands 1–3 (*p* < 0.0001 for each), Band 4 (*p* = 0.0015) and Band 5 (*p* = 0.0022). There was a significant sleep/wake effect in IS (*F* = 529.34, *p* < 0.0001), and IS during sleep was lower than in wake (*p* < 0.0001). There were significant band effects (*F* = 33.75, *p* < 0.0001) and sleep/wake periods (*F* = 38.37, *p* < 0.0001) on IV. The post hoc comparisons with Bonferroni correction revealed that the IV in Band 1 (10 to 30-min fluctuations) was higher than other bands (*p* < 0.0001).

**Table 2:** Parameters of circadian cosinor regression for coherence.

Band	Mean	Mesor	Mean amplitude	Acrophase
	Mean (SD)	Mean (SD)	Mean (SD)	Mean (SD)
1	0.245 (0.045)	0.053 (0.009)**	0.030 (0.023)	11:19 (6.2)
2	0.263 (0.060)	0.057 (0.012)**	0.035 (0.026)	10:21 (5.5)
3	0.260 (0.050)	0.057 (0.010)**	0.036 (0.027)*	11:29 (6.4)
4	0.285 (0.053)	0.061 (0.012)*	0.035 (0.024)	10:49 (6.3)
5	0.283 (0.069)	0.061 (0.014)*	0.034 (0.027)	12:09 (6.1)
6	0.324 (0.117)**	0.070 (0.025)	0.029 (0.028)	12:13 (6.0)
7	0.337 (0.151)**	0.071 (0.067)	0.025 (0.024)	12:32 (5.5)

Results of sex adjusted model.

In the sex and timing of meals adjusted model, mesor bands 4 and 5, and amplitude band 3 were no longer significant.

**p < .01; *p < .05.

In the second model (sex and meal timing adjusted), there was a significant band effect on the mesor (*F* = 8.51, *p* < 0.0001), and the post hoc comparisons with Bonferroni correction revealed that the Band 7 was significantly greater than Bands 1–3 (*p* < 0.0001, 0.0005, and 0.0005, respectively) and the Band 6 was significantly greater than Bands 1–3 (p < 0.0001, 0.0016, and 0.0016, respectively). There was a significant sleep/wake effect in IS (*F* = 493.68, *p* < 0.0001), and IS during sleep was lower than in wake (*p* < 0.0001). There were significant effects of band (*F* = 33.99, *p* < 0.0001) and sleep/wake periods (*F* = 36.45, *p* < 0.0001) on IV.

The mean acrophase (time of peak activity) was consistent across bands ranging from 10:35 a.m. to 12:30 p.m. (mean = 11:30 am). The mean phase delay did not differ among the band and sleep/wake period (*F* = 0.95, *p* = 0.463, and *F* = 008. *p* = 0.799, respectively) in the first and second models (sex adjusted), the mean phase delay did not differ among the band and sleep/wake period (*F* = 1.42, *p* = 0.207, and *F* = 0.03 *p* = 0.869, respectively). The sleep/wake with band interaction effect was not significant. The mean sleep mid-point was 4:11 am ± 1 h 11 min. A later acrophase was significantly associated with higher overall and nocturnal glucose variability (*J* index *r* = 0.356, *p* = 0.023 and *r* = 0.332, *p* = 0.034 respectively).

## Discussion

We systematically evaluated the relationship between routine unstructured activity and glucose variations across sleep and wake periods for one week in young adults with T1D in their home environment. Substantial state and circadian coupling between unstructured physical activity and glucose variations were observed, especially for the slower oscillation bands. There were significant circadian variations over the 7-day period for all circadian variables. Further, there were significant differences between sleep and wake, with higher mean activity and glucose coherence during sleep than wake. Based on our findings, we suggest that there are multiple modes of unstructured physical activity/glucose coupling.

Findings from the current study replicate and build on previous studies in adults with T1D and other populations. In the current study, we observed increased coherence strength with an increase in band period and a significantly higher mean coherence with the longest band (slowest oscillation), replicating Farabi et al.'s findings^4^. Further, we demonstrated the persistence of this relationship with unstructured activity across weekdays and weekend days over a 168-h period (compared to 60-h in the Farabi study).

Though there is a bidirectional relationship between unstructured physical activity and blood glucose in individuals with T1D^17^, glucose fluctuations alone play a role in sleep disruption in people with T1D. Naturally occurring hypoglycemia is associated with more frequent awakenings or movements during sleep in children, adolescents, and young adults with T1D^4,18,19^. Consistent with previous findings^4^, we observed a significantly higher mean coherence during sleep than wake. We found that the fluctuations in coherence between blood glucose in the shorter-duration bands were characterized by changes in blood glucose followed by changes in activity (supported in Farabi et al.'s study)^4^. We also found that a later acrophase (later time of peak activity) was associated with overall and nocturnal glucose variability in the current sample. These findings support the notion that those with T1D are at an elevated risk of nocturnal hypoglycemia following an evening or late afternoon session of structured activity of a high intensity or long duration as shown in experimental and observational studies^4,10,12,15^. These findings are important for insulin-glucose homeostasis considering sleep disruption from fragmented sleep results in decreased insulin sensitivity in adults without chronic conditions^20^. Taken together, the extent that glucose fluctuates as a result of sleep disruption may ultimately result in decreased insulin sensitivity, further inhibiting the ability to achieve glycemic targets in young adults with T1D.

Glucose fluctuations outside of range contribute to endothelial damage and the onset or progression of premature micro-and macrovascular complications in individuals with T1D^21^. Monocyte adhesion to the endothelium is enhanced in hyperglycemia^22–24^. Adherence of monocytes due to the up regulation of adhesion molecules on the endothelium and leukocytes are key processes in the development and progression of atherosclerosis^25^. Thus, identifying mechanisms by which these hyperglycemia-induced endothelial changes in T1D is a prime area needing further exploration to prevent or delay the onset of atherosclerotic cardiovascular disease (ASCVD). This is especially important as ASCVD is the leading cause of death and the life expectancy in T1D is approximately 12 years shorter than the general population^26^.

The significant coupling between glucose and unstructured physical activity exhibited a circadian pattern in the current study. These intrinsic time of day effects are, therefore to an extent, independent of the sleep or wake condition and have been demonstrated in experimental studies of adults without chronic conditions^3,27,28^ and in observational studies in those with T1D^4,29^. We found peak coherence between the parameters in the late morning/early afternoon (between 10:20 am and 12:30 pm) compared to late afternoon/early evening (between 5:15 pm and 6:30 pm) discovered in Farabi's study. These differences may be due to our longer measurement period and inclusion of weekend and weekdays. Additionally, based on previous studies of individuals with T1D, we suggest that while insulin sensitivity exhibits a diurnal pattern in this population, it is likely also specific to the individual^30^.

Interpreting the current study findings should be considered within the context of strengths and weaknesses. The current sample is well characterized with seven days, including week and weekend days of concurrent actigraphy and CGM. Those with a previous OSA diagnosis and those screened with a high-risk tool with a high negative predictive value for sleep apnea were screened out for exclusion, reducing but not eliminating an independent impact of abnormal rates of sleep-disordered breathing on glycemia. However, we did not use lab polysomnography, so there may still be participants with sleep apnea in the current sample. The study was cross-sectional from a single site, without a comparison group, and objective data on structured physical activity, melatonin, insulin dose, or other hormones (growth hormone, cortisol, etc.) were not collected. Incorporating a control group without a chronic condition and monitoring and controlling for internal and external zeitgebers (e.g., hormonal and insulin treatment effects) over a longer period (more than 1 week) may provide further insight into the findings presented here.

## Conclusion

Our findings suggest that both circadian rhythmicity and sleep play a role in the 24-h variation of glucoregulation. The 24-h variation in glucoregulation is intrinsically affected by the time of day independent of the sleep or wake condition (circadian) and intrinsically affected by the sleep condition irrespective of the time of day (sleep state). Thus, circadian rhythmicity and sleep are important physiological regulators of glucose, even in those with an absent autoregulation (T1D). Further studies are needed to delineate the respective roles of glucose production and utilization and the potential for improved meal and insulin timing to optimize glucose and sleep in this population reliant on exogenous insulin.

## Method

### Participants

This was a prospective observational single-center study conducted in the Northeastern United States. Participants were recruited from pediatric and adult diabetes clinics. We reported previously that better sleep and circadian characteristics were associated with better glycemia across 6–14 days of monitoring between persons^31^ and that poorer sleep (lower efficiency, longer wake after sleep onset) predicted higher next day glucose variability and vice versa within-person^32^. Young adults monitored their sleep and glucose patterns concurrently for 6–14 days with a sleep–wake activity monitor (Phillips Respironics Spectrum Plus) and either their continuous glucose monitor (CGM) or a provided blinded Dexcom G4. The CGM was blinded to observe the sleep-glucose relationship in a naturalistic setting, considering the introduction of a CGM may lead to intervention effects. Time-varying coherence (a measure of the correlation between two signals) in sleep and glucose patterns using the actigraphy and CGM data were analyzed.

After participants were determined eligible based on chart review, they were screened for inclusion over the phone using the Berlin Questionnaire^33^. We referred those at high risk for sleep apnea for treatment and did not include them in the study. Young adults with T1D for at least 6 months (to avoid the initial confounding effect of endogenous insulin section) with no other major medical or psychiatric comorbidity, who wore or were willing to wear a CGM, did not have a previous OSA diagnosis, were not pregnant, and did not work night shifts participated in this longitudinal study in the natural environment.

We followed the World Medical Association Declaration of Helsinki for research standards involving human subjects^34^. The study was approved by the Case Western Reserve University Institutional Review Board (#20200650). Informed consent was obtained from participants before collecting data.

### Actigraphy

Research grade actigraphy provides comparable estimates of sleep parameters compared to polysomnography and has been validated in young adults with T1D^35^. Thus, actigraphy is a tool clinicians and researchers can use to examine sleep and circadian patterns through an economic non-invasive home assessment over an extended period of time^36^. Participants were instructed to continuously wear the Spectrum Plus on their non-dominant wrist continuously for 7–14 days and depress the event marker at "lights out" and "lights on" times to demarcate time in bed.

### Glucose

Young adults wore their own or a provided CGM blinded (Dexcom G4) to measure glucose levels every 5 min throughout the monitoring period (up to 288 readings in a 24-h period). CGMs were worn concurrently with the actigraph. CGM data were downloaded directly to capture glucose patterns. Participants without a CGM inserted a small sensor wire in the abdominal subcutaneous tissue using an automatic inserter during the baseline study visit^37^. Participants were instructed to calibrate the Dexcom G4 every 12 h with a finger stick.

### Wavelet coherence analysis (WCA)

WCA was used to establish each participant's concurrent unstructured physical activity and glucose variations in each band. The actigraph records activity data in 30-s epochs, whereas the CGM collects the data every 5 min (up to 288 times per day). The CGM and actigraph data were aligned by date and time, and the actigraph data were aggregated every 5-min using Python 3.5^38^. Mean imputation was used to handle missing data for CGM and actigraphy in Python 3.5^38^ for WCA.

WCA allows for determining both dominant modes of variability and how these modes vary in time^19^. WCA finds regions in time–frequency space where the two-time series co-vary^18,19^. We used the Morlet wavelet function in MatLab R2022a, performing computations with the wavelet toolbox developed by Grinsted 2004^18^. This function yielded coherence values every 5 min for each of the 96 oscillations, with periods ranging from 15 to 3,281 min. For the present analysis, we excluded all values potentially influenced by "end effects," as described by Grinsted, Moore, and Jevrejeva (2004), and to replicate previous findings from Farabi, Carley, and Quinn^4^.

The wavelet coherence of two-time series *x* and *y* is:

$$\frac{{\left| {S\left( {C_{x}^{*} \left( {a,b} \right)C_{y} \left( {a,b} \right)} \right)} \right|^{2} }}{{S\left( {\left| {C_{x} \left( {a,b} \right)} \right|^{2} } \right) \cdot S\left( {\left| {C_{y} \left( {a,b} \right)} \right|^{2} } \right)}}$$

To facilitate interpretation of the 168-h (7-day) recordings, we collapsed the 96 wavelet oscillations into 7 bands with different period ranges (Band 1, 15–45 min; Band 2, 45–90 min; Band 3, 90–180 min; Band 4, 180–360 min; Band 5, 360–720 min; Band 6, 720–1440 min; and Band 7, 1440–2880 min). We also segmented each recording according to sleep and wake periods, yielding seven approximately 8-h (5 h 57 m to 10 h 12 m) segments for each circadian day.

### Circadian analysis

To characterize circadian variations, we used cosinor analysis. We fitted a cosine wave using least squares regression for each band with a period of 1,440 min (24-h) to the coherence data as follows:

$$Y_{i} \left( t \right) = M_{i} + A_{i} \cdot \cos \left( {\frac{2\pi t}{\tau } + \varphi_{i} } \right) + e_{i} \left( t \right),$$

where the three specific rhythmometric parameters represent the $M_{i}$, mesor, The Midline Estimating Statistic of Rhythm; $A_{i, }$ the amplitude; and $\varphi_{i}$, the acrophase. The mesor is a rhythm-adjusted mean of activity movements over a 24-h period, with higher values representing more robust movement. The amplitude represents half of the predicted extent of rhythmic change. The acrophase represents the time interval within the highest recorded values for participant *i* respectively and indicates the peak alertness time^39,40^. For the 24-h cycle of the rhythm, the period ($\tau$) is 1,440 min (60 min/hours × 24 h). Those parameters were estimated with the least-squares approach^40^.

The timing of the peak activity ($\emptyset$) was calculated with the equation $\emptyset = \left( { - \frac{24}{{2\pi }}} \right) \cdot \varphi$.

We report the amplitude, acrophase (clock time of peak coherence) means, and Pearson correlation (r^2^) for each band. Two non-parametric parameters (interdaily stability (IS) and intradaily variability (IV) were included from the wrist actigraph recordings. The IS provides an estimate of how closely the 24-h rest-activity rhythm follows the 24-h light–dark cycles, while IV provides an estimate of the fragmentation of the 24-h rest-activity rhythm^41^.

### Statistical analysis

We used a repeated-measures linear mixed model with a compound symmetric covariance structure adjusted for sex using the SAS Proc Mixed procedure to identify differences in coherence parameters among the bands and between sleep/wake intervals using each of these factors as a repeated measure (SAS 9.4, SAS Institute, Cary, NC, USA). Next, we added timing of meals into the model. First, we added each meal separately into the model, and the results were not significant (data not shown). Next, we incorporated dinner timing into the second set of models (sex and timing of meals). Finally, we determined pairwise differences between bands and sleep/ wake intervals by post hoc comparisons controlled by Bonferroni correction.
